# NPM1 Silencing Reduces Tumour Growth and MAPK Signalling in Prostate Cancer Cells

**DOI:** 10.1371/journal.pone.0096293

**Published:** 2014-05-05

**Authors:** Gaëlle Loubeau, Rafik Boudra, Sabrina Maquaire, Corinne Lours-Calet, Claude Beaudoin, Pierre Verrelle, Laurent Morel

**Affiliations:** 1 Clermont Université, Université Blaise Pascal, GReD, Clermont-Ferrand, France; 2 CNRS, UMR 6293, GReD, Clermont-Ferrand, France; 3 Inserm, UMR 1103, GReD – Genetics, Reproduction and Development, Clermont-Ferrand, France; 4 CRNH, Centre de Recherche en Nutrition Humaine, Clermont-Ferrand; 5 Clermont Université, Université d'Auvergne, EA 7283 CREaT -Cancer Resistance Exploring and Targeting, Clermont-Ferrand, France; 6 Centre Jean Perrin, Clermont-Ferrand, France; Thomas Jefferson University, United States of America

## Abstract

The chaperone nucleophosmin (NPM1) is over-expressed in the epithelial compartment of prostate tumours compared to adjacent healthy epithelium and may represent one of the key actors that support the neoplastic phenotype of prostate adenocarcinoma cells. Yet, the mechanisms that underlie NPM1 mediated phenotype remain elusive in the prostate. To better understand NPM1 functions in prostate cancer cells, we sought to characterize its impact on prostate cancer cells behaviour and decipher the mechanisms by which it may act. Here we show that NPM1 favors prostate tumour cell migration, invasion and colony forming. Furthermore, knockdown of NPM1 leads to a decrease in the growth of LNCaP-derived tumours grafted in Nude mice *in vivo*. Such oncogenic-like properties are found in conjunction with a positive regulation of NPM1 on the ERK1/2 (Extracellular signal-Regulated Kinases 1/2) kinase phosphorylation in response to EGF (Epidermal Growth Factor) stimulus, which is critical for prostate cancer progression following the setting of an autonomous production of the growth factor. NPM1 could then be a target to switch off specifically ERK1/2 pathway activation in order to decrease or inhibit cancer cell growth and migration.

## Introduction

The progression of prostate cancer is associated with alterations of key genes that control the cell homeostasis, their deregulation and/or amplification leading to increased cell proliferation and invasive capacities. We previously identified *NPM1* (nucleophosmin 1) as one of the genes whose expression is significantly increased in prostate tumour cells when compared to non-tumour adjacent tissue [1], indicating that NPM1 could act as an enhancer of prostate cancer progression. NPM1 is a major multifunctional phosphoprotein accumulated at high level in the granular region of the nucleolus and is able to shuttle between the nucleolus, the nucleoplasm and the cytoplasm [2]. Because of its nucleolar localization, its intrinsic RNase activity and its association with maturing pre-ribosomal ribonucleoproteins, NPM1 has been first proposed to regulate ribosomal RNA transcription and processing. However, NPM1 has been more recently demonstrated to display chaperone activities. It binds to histones, favours DNA-histone assembly, mediates nucleosome formation and relaxes chromatin [3] thereby controlling gene expression. NPM1 also interacts with a wide range of maturating proteins to induce their proper folding in the active state. Among those proteins, there are cell growth regulators such as the oncoprotein MDM2 (Mouse Double Minute 2 homolog). Furthermore, NPM1 binds to and inhibits the tumour suppressor proteins P53 and Rb (Retinoblastoma) [4] highlighting that NPM1 could have a role in oncogenic processes. Some of the NPM1 specific interactions with cell cycle regulators have already been clarified, but its role in the behaviour of solid tumour cells, as well as its integration in the cell signalosome is yet to be determined. Here we address the question whether NPM1 could potentiate proliferation, migration and invasion capacities of prostate cancer cells. In this study, we report that the level of NPM1 in prostate cancer cells specifically regulates EGF expression and the MAPK (Mitogen Activated Protein Kinases) signalling pathway. We also show that high levels of NPM1 positively impact cell proliferation and cell migration, thus participating in the control of tumour growth.

## Materials and Methods

### Ethics statement

All animals were maintained in a controlled environment and animal care was conducted in compliance with the national standard policies (C 63 014.19). All experiments were approved the Auvergne Regional Ethics Committee, France (protocol CE09-08).

### Cell culture and stable transfection

LNCaP (Lymph Node Carcinoma Prostate) cells were cultured in phenol red Roswell Park Memorial Institute 1640 medium (RPMI 1640, Life Technologies, Saint-Aubin, France) supplemented with 10% heat-inactivated fetal bovine serum (FBS) and incubated in standard conditions (37°C, 5% CO_2_).

Cells were infected according to manufacturer's instructions with lentiviral particles containing either three target-specific constructs (shNPM1) or unrelated sequences (shScr, Srambled) (sc-29771-V, Santa Cruz, Heidelberg, Germany). Following infection, puromycine (1 µg/ml) was added to the culture medium in order to select stably transduced cells and to perform monoclonal selection.

### Wound healing migration assay

Control LNCaP cells (shScr) and NPM1 knocked-down LNCaP cells (shNPM1) were seeded in 24-wells plates and grown to confluence for 24 hours. The monolayer culture was then scrape-wounded with a sterile micropipette tip in order to create a gap of constant width. Cellular debris were washed with Phosphate Buffered Saline 1× (PBS) (Life Technologies). Cells were next grown in RPMI 1640 10%FBS that was replaced 12 hours after wounding and then every 24 hours. LNCaP cell migration was photographed into the wounded region at 24, 48 and 72 hours following the scraping (100× magnification) and remaining wound areas were then quantified with ImageJ free software.

### Boyden Chamber invasion assay

For cell migration assay, 3×10^5^ shScr and shNPM1 LNCaP cells cultured in serum free RPMI 1640 were seeded into the upper well of a transwell chamber system. Medium containing 10% FBS was added to the lower chamber. After incubation for 24 to 48 hours, the non-migrated cells were removed with the upper well. The cells that migrated to the bottom insert surface were then fixed with methanol and stained with a 5% Giemsa solution. Five random fields were photographed (200× magnification) and cells were quantified with ImageJ free software as the mean ± SD of colonies counted per field.

### Soft agar colony formation assays

Six-wells plates were prepared with 2 ml/well of a warm solution of 0.6% low melting agarose (Agarose Sea Plaque FMS product low melting, 50101, LONZA, Ozyme, Montigny-le-Bretonneux, France) in RPMI 1640, 10%FBS. After solidification, 5×10^3^ of shScr or shNPM1 LNCaP cells were plated per well in a solution of 500 µl of 0.3% agarose in RPMI 1640 medium containing 10%FBS. The plates were then incubated in RPMI 1640, 10%FBS medium that was changed every 3–4 days. After 2 weeks, colony formation was quantified using the ImageJ free software: colonies were photographed (100× magnification) on 5 different fields and the relative colony number was calculated as the mean ± SD of the colonies counted per field.

### Clonogenic assay

1×10^4^ shScr and shNPM1 LNCaP cells were seeded in 6-wells plates in RMPI 1640, 10%FBS medium. Medium was changed every 3 days. After 2 weeks, the medium was removed and cells were washed with PBS 1× then fixed with methanol and stained with a 5% Giemsa solution. Foci formation was evaluated and colony formation was quantified using the ImageJ free software: colonies were photographed (100× magnification) on 5 different fields and the relative colony number was calculated as the mean ± SD of the colonies counted per field.

### Cell proliferation assay

Cell proliferation was determined using Cell proliferation ELISA BrdU (Bromodéoxyuridine) colorimetric kit (Roche) according to the manufacturer's instructions. Briefly 5×10^3^ shScr or shNPM1 LNCaP cells per well were seeded in a 96-wells plate. They were cultured for 24 hours in RPMI 1640 medium, 10% FBS in the presence of absence of EGF (E9644, Sigma). Then, BrdU labelling solution was added at a final concentration of 10 µM for 2.5 hours. After fixation, the cells were incubated with anti BrdU-POD solution for 1 hour, and absorbance was measured at 655 nm.

### Western Blotting

Proteins were extracted using HEPES 20 mM, NaCl 0.42 M, MgCl_2_ 1.5 mM, EDTA 0.2 mM, and Nonidet P-40 1% supplemented with phenylmethylsulfonyl fluoride (PMSF) 1 mM (Sigma), protease inhibitors (Complete 1X; Roche), NaF 0.1 mM, and Na_3_VO_4_ 0.1 mM (Sigma). Forty µg of total proteins were then subjected to denaturing SDS-PAGE and transferred to nitrocellulose Hybond-ECL membrane (GE Healthcare Life Sciences, Velizy-Villacoublay, France). Detections were performed using antibodies raised against β-actin (A2066, Sigma), NPM1 (sc-6013-R, Santa Cruz, Heidelberg, France), EGFR (Epidermal Growth Factor Receptor, NB100596, Novus), phospho-EGFR Tyr 1068 (2236S, Cell Signaling, Ozyme), ERK1/2 (M5670, Sigma, Saint-Quentin Fallavier, France), phospho-ERK1/2 p42/44 (9101S, Cell Signaling, Ozyme) AKT (9272, Cell signaling, Ozyme), phospho-AKT Ser 473 (2118-1 Epitomics) and revealed with peroxidase-conjugated anti-rabbit IgG (Immunoglobulin G, P.A.R.I.S, Compiègne, France) using a Western Lightning System kit (PerkinElmer, Villebon sur Yvette, France). Signal quantification was performed using Quantity One software (Bio-Rad).

### Promoter construct, transfection and luciferase assays

The human EGF promoter fragment (−392/+123) was generated with the following primers: 5′-GATCAAGCTTGGGCTGAAGGTGAACTATCTTTAC-3′ (Forward) and 5′-GATCCTCGAGGACAGAGCAAGGCAAAGGCTTAGA-3′ (Reverse), then cloned into the HindIII/XhoI restriction sites in a pGL3-basic vector (Promega, Charbonnieres, France). The constitutively active MAP kinase kinase kinase1 (caMEKK1) expression plasmid was a kind gift from Dr. Dirck Bohmann (University of Rochester Medical Center, Rochester, USA). shScr and shNPM1 LNCaP cells were transfected 24 h after seeding with the pGL3-hEGF and/or caMEKK1 plasmids in OPTI-MEM using Metafectene transfectant according to the manufacturer's instructions (Biontex, Martinsried-Planegg, Germany). Twenty-four hours after transfection, cells were lysed into Reporter lysis buffer 1× (Promega) and the luciferase activity was measured using the Genofax A luciferase assay kit (Yelen, Ensue la Redonne, France).

### Preparation of RNA and real-time quantitative PCR

Total cellular RNA was extracted using TRIzol reagent (Life Technologies) and cDNA was synthesized with 200 U of Moloney murine leukemia virus-reverse transcriptase (Promega), 5 pmol of random primers (C1181, Promega), 40 U RNAsin (Promega), and 2.5 mM deoxynucleotide triphosphate. mRNA levels were quantitated on a Mastercycler ep Realplex (MasterCycler2, Eppendorf, Le Pecq, France) with Mesagreen QPCR Mastermix Plus for SYBR (Promega). Sequences of the primers are the following: NPM1, 5′-ATGGAAGATTCGATGGACATGG-3′ (Forward) and 5′-CGAGAAGAGACTTCCTCCACTGC -3′ (Reverse); PCNA, 5′-TGCCTTCTGGTGAATTTGCACGT-3′ (Forward) and 5′- ACCGTTGAAGAGAGTGGAGTGGC-3′ (Reverse), Actin, 5′-CGCGAGAAGATGACCCAGATC-3′ (Forward) and 5′TCACCGGAGTCCATCACGA-3′ (Reverse) (Eurogentec, Angers, France). For human EGF, primers were purchased from Qiagen (PPH00137B-200, Qiagen, Courtaboeuf, France).

### *In vivo* Nude mouse tumour xenograft model

ShScr and shNPM1 LNCaP cells were grown to confluency then resuspended in matrigel (BD matrigel basement membrane matrix phenol red free, BD Biosciences, Le Pont de Claix, France) to a concentration of 9×10^6^ cells/ml. Three hundred µl of the cell suspension (approximately 3×10^6^ cells) were injected subcutaneously in 6 weeks old Nude mice (Swiss NU/NU, Charles River, L'Arbresle, France). Disease progression was monitored daily, based on a set of general wellness criteria set by the animal care committee, and body mass was recorded thrice a week until a predetermined endpoint was reached. Endpoints included: dehydration and/or weight loss of over 10%, any evidence of respiratory distress, body weight increase of over 5 g from the average. Tumours were measured thrice a week using an electronic caliper since palpable tumours were detectable. Mice were killed by cervical dislocation under gas anesthesia. Xenografts were then removed, weighted and snap-frozen in liquid nitrogen for RNA and protein analyses.

### Statistical Analysis

All assays were done in at least 3 independent experiments. Standard errors were calculated for each mean, and statistical differences between groups were determined by Student's *t* test or the non-parametric Mann & Whitney U test using Prism software. For longitudinal analyses, a random-effect model (mixed model) was considered to study the fixed effects group (NPM1 expression), time-points and interaction group × time taking into account between and within subject variability (random effects slope and intercept).

## Results

### NPM1 regulates clonogenic and proliferative capacities of prostate tumour cells

**Figure 1:**
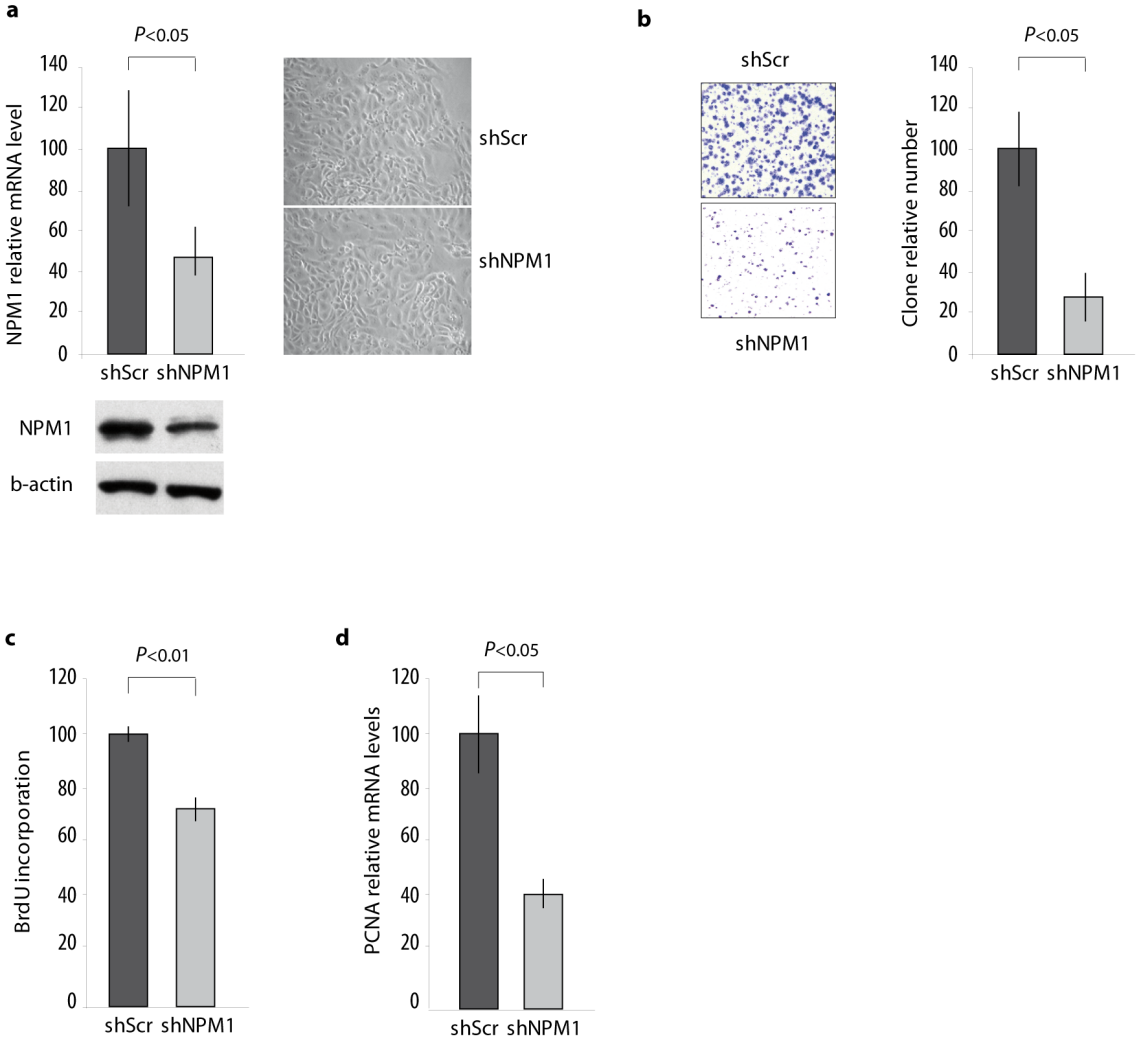
Regulation of LNCaP cell clonogenic capacities and proliferation rate by NPM1. (**a**) NPM1 knockdown does not alter LNCaP cells morphology. LNCaP cells were stably transfected with control shRNA (shScr) or specific NPM1 shRNA (shNPM1). NPM1 mRNA and protein levels were analysed by RT-qPCR and western blotting respectively. Morphology of cells was observed by inverted microscopy and photographed. (**b**) NPM1 knockdown inhibits LNCaP cells clonogenicity. Cells were seeded at low confluence over one week, then fixed with methanol and stained with 5% Giemsa blue before microscopic observation. Pictures are representative of three independent experiments with consistent results. The graph represents the number of cell clones (>50 cells) in the shNPM1 condition, calculated as the mean ± SD of the number of clones counted per field, on 5 random fields, using the ImageJ free software and expressed relatively to the number of clones counted in the control condition. (**c, d**) NPM1 controls proliferation of prostate cancer cells. Five thousand cells were seeded per well in a 96-wells plate and cultured for 48 hours. (**c**) Cells were then incubated with a BrdU labeling solution for 2.5 hours and BrdU incorporation was measured by densitometric analysis at 655 nm. (**d**) Proliferation was also analysed by RT-qPCR assay by evaluating PCNA relative mRNA level accumulation normalized using β-actin mRNA level. All data are representative of at least three independent triplicate experiments and BrdU incorporation as mean of triplicate experiments of 96 points each. Data are expressed as the mean ± SD.

In order to analyse the impact of NPM1 on prostate tumour cell proliferation, we chose a knockdown strategy and generated LNCaP cell sublines stably expressing control (shScr) or NPM1 specific shRNA (shNPM1). As shown in figure 1a, NPM1 mRNA level decreases by more than 50% and NPM1 protein expression by more than 30% in shNPM1 cells, but this is not associated with modifications in cell morphology. However, NPM1 knockdown alters the ability of LNCaP cells to form colonies after seeding at low confluency (figure 1b). This decrease of clonogenic capacities coincides with a decrease in the cell proliferative potential (figure 1c). Indeed, the down-regulation of NPM1 leads to a 30% decrease in BrdU incorporation and this is associated with a 60% decrease in the mRNA level of PCNA (Proliferating Cell Nuclear Antigen), a key marker of cell proliferation (figure 1d). Interestingly, NPM1 knockdown does not alter cell survival as evaluated by PARP (Poly (ADP-ribose) polymerase) cleavage analyses by western blotting (figure S1), thus indicating that this decrease in the proliferation rate is not associated with an increase of apoptosis. These results consequently show that NPM1 is involved in the increase of proliferation and clonogenic capacities of prostate tumour cells.

### NPM1 impact on proliferation is associated with the control of migration, invasion, three-dimensional growth capacities of prostate cancer cells and tumour growth

**Figure 2:**
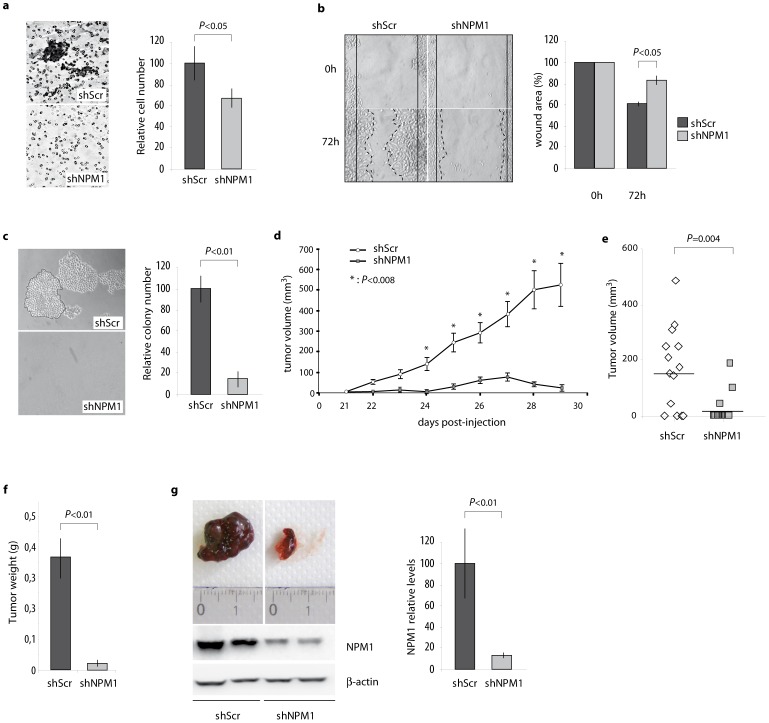
NPM1 knockdown impacts migration, invasion and growth of prostate cancer cells. (a) NPM1 controls migration capacities of LNCaP cells. Cells were seeded at confluence in order to create a wound 24 hrs later. Wound recolonization was observed after 72 hours of culture by inverted microscopy and photographed. Histograms show wound area quantification using the ImageJ and pictures are representative of three independent experiments with consistent results. (**b**) NPM1 knockdown inhibits the invasive potential of prostate cancer cells. shScr and shNPM1 LNCaP cells were seeded at confluency in RPMI 1640 serum free medium on matrigel coated microporous membrane. 48 hrs later, migrated cells present on the opposite side of the membrane were fixed and stained with 5% Giemsa blue and observed at microscope (200× magnification). The graph represents the number of cells in the shNPM1 condition, calculated as the mean ± SD of the number of cells counted per field, on 5 random fields, using the ImageJ free software and expressed relatively to the number of cells counted in the control condition. (**c**) NPM1 impacts three-dimensional growth of prostate cancer cells. Control or NPM1 knocked down cells were seeded at low confluency on agarose/RPMI 1640 10% FBS for 2 weeks. Number and size of the emerging clones were then observed under inverted microscope (x100) and photographed. The graph represents the number of cell clones (>50 cells) in the shNPM1 condition, calculated as the mean ± SD of the number of clones counted per field, on 5 random fields, using the ImageJ free software and expressed relatively to the number of clones counted in the control condition. (**d, e, f,g**) NPM1 knock-down abrogates tumourigenicity of LNCaP cells when injected in nude mice. shScr (n = 14) and shNPM1 (n = 14) LNCaP cells were subcutaneously grafted on nude mice and tumour volume was measured every 2 days following engraftment (**d**). Graph in (**e**) is the quantitation of shScr and shNPM1-derived tumours volume at day 24 post-injection. NPM1 relative expression level was evaluated by western blotting in tumours when mice were sacrified (f) and tumour weight was measured (g). The data are representative of at least three independent experiments and are expressed as the mean ± SD.

The above results demonstrate that NPM1 exerts a control on the clonogenic capacities of prostate cancer cells and suggests that, besides its effect on the cell proliferative rate, it could also contribute to both tumour growth and aggressiveness. To further evaluate the role of NPM1, we examined the invasion and migration capacities of shNPM1 LNCaP cells. The knockdown of NPM1 significantly decreased the invasion of LNCaP cells through matrigel-coated filters by 40% (figure 2a). We further assessed the LNCaP cells migration ability by physically wounding cells plated on the cell culture plates. As shown in Figure 2b, at 72 h after being scrubbed, shNPM1 cells were unable to recolonize denuded zone as faster as did the control cells. To test whether the decrease of migration and invasion capacities is accompanied by a decrease of the three-dimensional growth abilities of shNPM1 LNCaP cells, *in vitro* tests in soft agar were performed (figure 2c). Decrease in NPM1 in LNCaP cells almost abolishes their ability to form 3-D colonies. These results from *in vitro* assays strongly suggest that NPM1 regulates the migratory and invasive properties of prostate cancer cells.

To further determine the role of NPM1 in prostate cancer, we analysed tumourigenesis of the shNPM1 LNCaP and shScr LNCaP prostate cancer cell lines *in vivo* following subcutaneous injection in the flank of male nude mice. ShNPM1 cells produce smaller tumours (figure 2d,e) and, contrary to shScr LNCaP cells, are more likely to produce no tumour at all when grafted (figure 2e). In detail, tumours initiated from LNCaP control cells appear at 22 days post-injection whereas tumours originating from LNCaP shNPM1 cells are noticeable only at 24 days post-injection. Furthermore, growth curves never get paralleled even after 29 days (figure 2d). Thus, tumours initiated from NPM1 knocked-down cells remained smaller than control tumours with 85% decrease of the average tumour volume (figure 2e). This important decrease of tumour volume results in a 95% decrease of tumour weight from LNCaP shNPM1 cells (figure 2g). This decrease is unlikely due to a loss of expression of the anti-NPM1 shRNA since all shNPM1 tumours preserve a 90% decrease in NPM1 accumulation (figure 2f). Taken together, these data clearly demonstrate that NPM1 is involved in prostate tumour growth *in vitro* and *in vivo*.

### NPM1 is involved in the control of EGF expression

**Figure 3:**
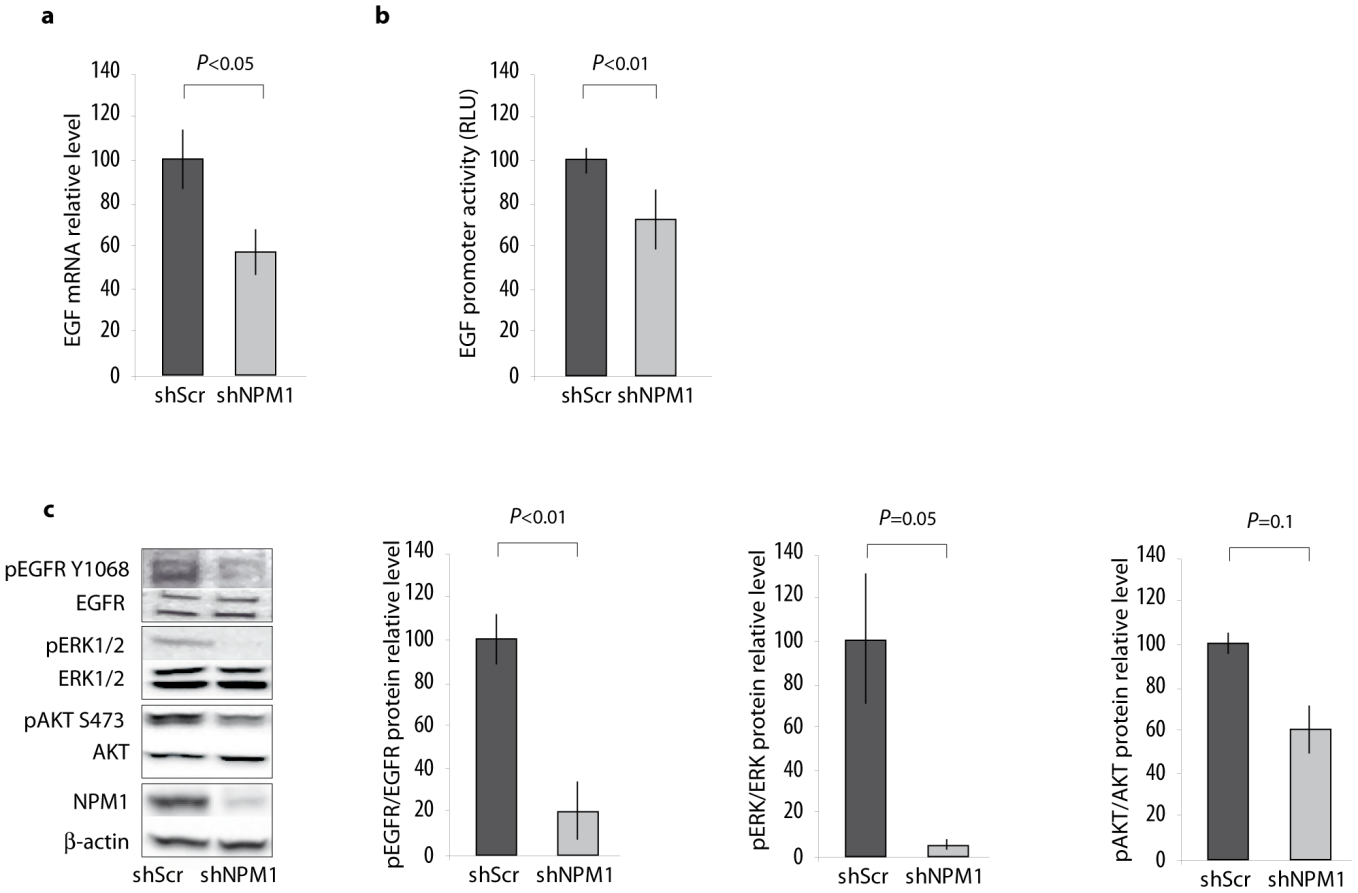
NPM1 knockdown decreases EGF expression. (**a**) NPM1 controls EGF expression. Relative EGF mRNA levels compared to β-actin were analyzed by RT-qPCR in LNCaP cells expressing control (shScr) or NPM1 specific shRNA (shNPM1). (**b**) NPM1 control EGF promoter activity. shScr and shNPM1 LNCaP cells were transfected with the phEGF-luciferase reporter plasmid. EGF promoter activity was evaluated by measuring the luciferase activity 24 hours later. Results of the assay were standardized using the CMV promoter as control and expressed as fold-induction over control cells (shScr). (**c**) NPM1 controls activation of the EGF/EGFR pathway downstream effectors. Proteins, extracted from shScr and shNPM1 LNCaP cells cultured in RPMI 1640 10%FBS, were electrophoresed by SDS-PAGE. Transferred membranes were immunoblotted with indicated antibodies. Histograms show the band quantification reported to the β-actin level. Blots are representative of three independent experiments with consistent results. Data are representative of at least three independent experiments and are expressed as the mean ± SD.

In order to better understand how NPM1 can promote prostate cancer cell behaviour, we performed a qPCR Array analysis (RT2 ProfilerTM array, PAHS-121A-2, Qiagen) and determined the nature of genes whose transcription levels are modulated by the knockdown of NPM1 (data not shown). Among the 84 genes spotted on the array, the Epidermal Growth Factor (EGF) mRNA accumulation was the most significantly decreased in LNCaP shNPM1 cells. This modulation of EGF expression does not result from a global inhibitory effect on gene expression as most of the genes are unaffected or up-regulated when NPM1 is decreased (data not shown). This result was confirmed by RT-qPCR assays as EGF mRNA accumulation is decreased by 40% in LNCaP shNPM1 cells (figure 3a). Moreover, to test the relative activity of the EGF promoter's gene activity in LNCaP shSCR and shNPM1 cells, we transfected these cells with a phEGF-luciferase reporter plasmid. Knockdown of NPM1 induces a decrease in EGF promoter activity, showing that the effect of NPM1 is likely to be exerted at the transcriptional level (figure 3b). These results suggest that NPM1 controls directly or indirectly EGF expression. As EGF is known to specifically activate the EGF receptor (EGFR), we investigated whether the activation of the EGFR complex as well as the activation of its downstream effectors, ERK1/2 and AKT, is modulated by NPM1 expression level. Based on the analysis of their phosphorylation status, we show that the activation of EGFR (pEGFR) and of ERK1/2 (pERK1/2) is dramatically decreased or almost abolished when the expression of NPM1 is inhibited (figure 3c). Interestingly, AKT phosphorylation is less sensitive to such an inhibition, suggesting that NPM1 specifically impacts the MAPK pathway (figure 3c). These effects of NPM1 on EGF expression and ERK1/2 pathway strongly suggest that NPM1 could be involved in the setting of the EGF self-regulation loop.

### NPM1 specifically potentiates the MAPK pathway activity to promote proliferation and migration capacities of prostate cancer cells

The above data demonstrate that NPM1 is involved in the control of EGF expression, a growth factor that controls the MAPK pathway and promotes tumorigenic behaviour of prostate cancer cells. So, we wondered if it was the reason why shNPM1 LNCaP cells were showing decreased tumorigenic behaviour. We thus investigated whether an exogenous intake of EGF could rescue the activation of the EGF/EGFR pathway effectors and subsequently increase LNCaP cells proliferation and migration. Control (shScr) or NPM1 knocked down (shNPM1) LNCaP cells were starved in order to switch off the signalling pathways and then treated with EGF.

**Figure 4:**
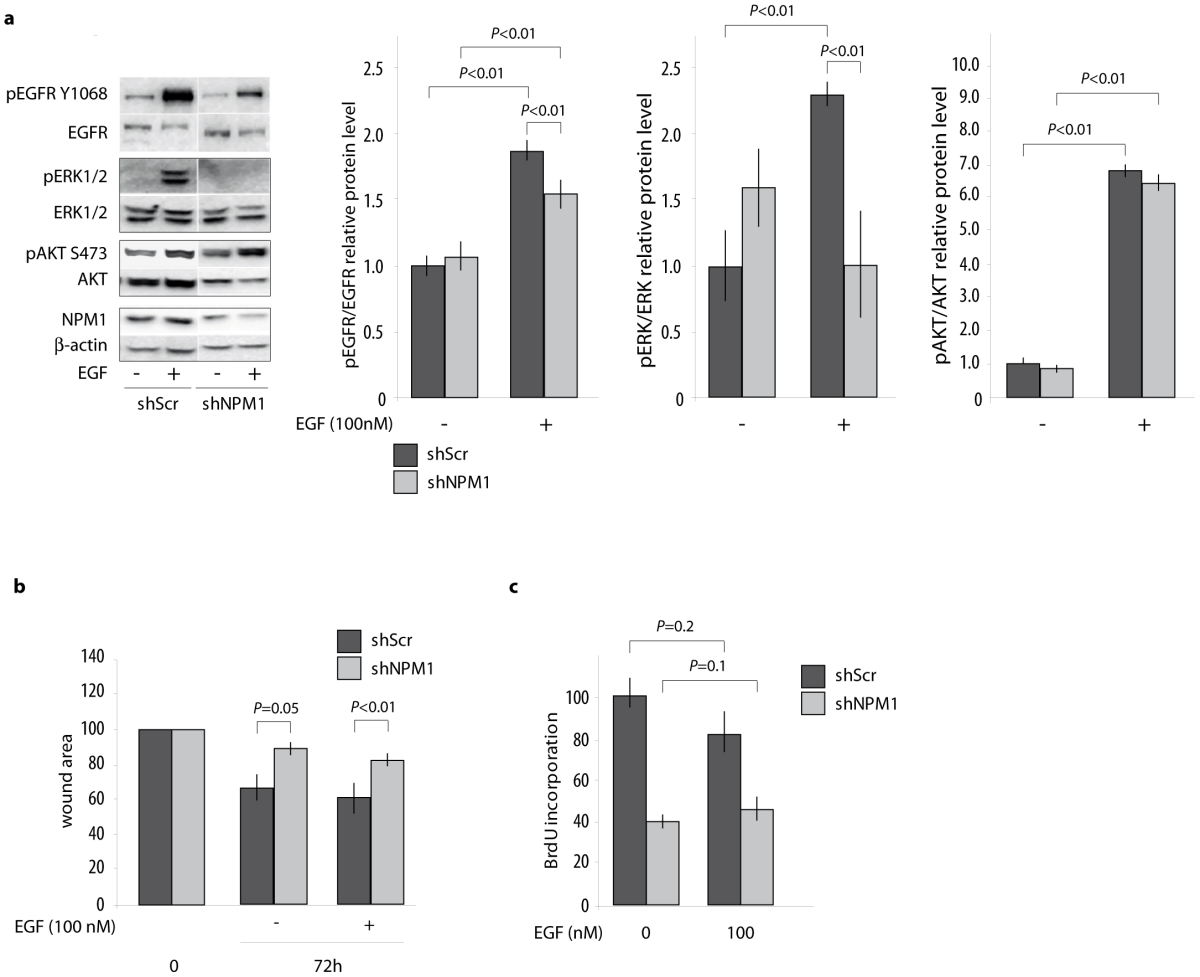
NPM1 knockdown in prostate cancer cells reduces proliferation and migration capacities by inhibiting the EGF/EGFR pathway activity. (a) NPM1 down-regulation inhibits EGF induced ERK1/2 pathway activity. shScr and shNPM1 LNCaP cells were treated with 100 nM EGF and phosphorylation of the EGF/EGFR pathway effectors was analysed using Western Blotting. Histograms show the band quantification reported to the β-actin level. The blot is representative of three independent experiments with consistent results. (b) Despite EGF treatment, migration capacities of LNCaP decreased for NPM1 were not restored. Wound closure was analysed 72 hours after continuous treatment with 100 nM EGF. Cells were observed under inverted microscope and photographed. Histograms show wound area quantification using ImageJ. (c) EGF treatment does not rescue proliferation of NPM1 knockdown LNCaP cells. BrdU incorporation assay was performed in shScr and shNPM1 LNCaP 24 hours after 100 nM EGF treatment. Densitometry was measured at 655 nm. The data are representative of at least three independent experiments and are expressed as the mean ± SD.

Western blot analyses show that an exogenous EGF supplementation leads to the phosphorylation, *i.e.*, the activation of both the EGF receptor and one of its downstream target, AKT, in both shScr and shNPM1 cells. On the contrary, and most interestingly, phosphorylation of ERK1/2 is specifically impaired in shNPM1 cells (figure 4a). In agreement with this result, addition of exogenous EGF is unable to restore migration and invasion capacities of shNPM1 LNCaP cells in the corresponding assays (figure 4b,c). These results clearly demonstrate that NPM1 is specifically required for the activation of the MAPK signalling pathway and that the potentiation of this transduction pathway is involved in the control of proliferation and migration capacities of prostate cancer cells.

### NPM1 acts upstream of MEK1 and downstream of EGFR to activate the MAPK pathway, for the control of an EGF self-regulation loop in prostate cancer cells

**Figure 5:**
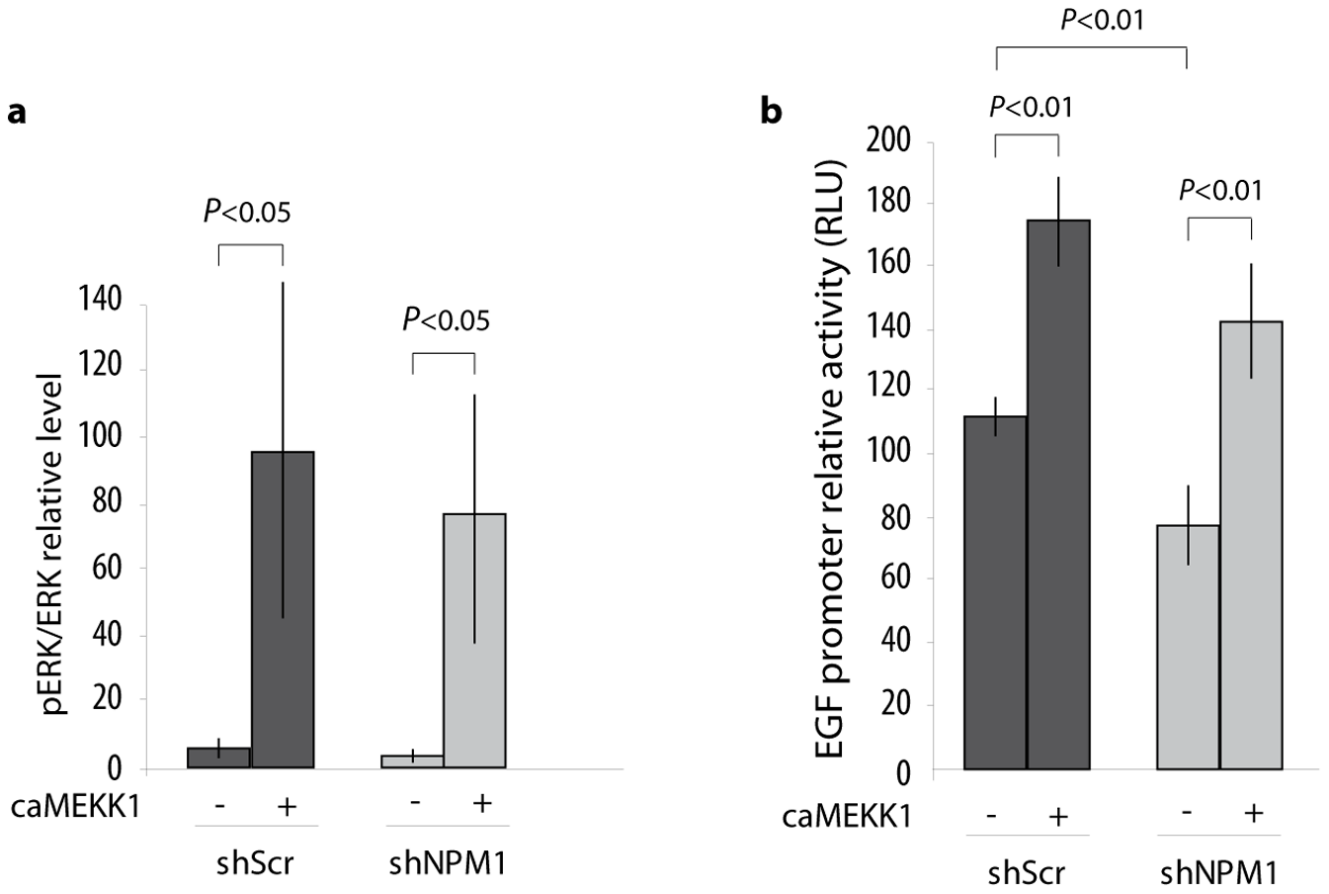
NPM1 does not act directly on EGF expression but upstream of the MEKK effector to activate the MAPK pathway in prostate cancer cells. (a) ERK1/2 activation is rescued by caMEKK1. shScr and shNPM1 LNCaP cells are transfected with a constitutively activated construct of MEKK1 (caMEKK1) and ERK1/2 phosphorylation level was analysed using western blotting. (b) ERK1/2 pathway activation restores EGF promoter activity in LNCaP cells downregulated for NPM1. LNCaP shSCR and shNPM1 cells were transiently co-transfected with phEGF-luc and caMEKK1. EGF promoter activity was evaluated by measuring the luciferase activity 24 hours after. Results of the assay were standardized against control reporter activity CMV-Luc and expressed as fold-induction over control cells (shScr). Data are representative of at least three independent experiments and are expressed as the mean ± SD.

As phosphorylation of ERK1/2 is specifically impaired in shNPM1 cells even in the presence of EGF, it suggests that, in the MAPK pathway, NPM1 acts upstream of ERK1/2. To identify the effector(s) of the EGFR-induced signalling pathway on which NPM1 may act, and to determine whether NPM1 action is direct or not on the EGF gene promoter, we performed transfection assays with a constitutively active form of MEKK1 (caMEKK1): caMEKK1 phosphorylates ERK1/2 kinase MEK1/2, and thereby, constitutively activates ERK1/2 in the absence of MEK1/2 inhibition. In shScr LNCAP cells, caMEKK1 transfection induces the phosphorylation of ERK1/2. In shNPM1 LNCaP cells caMEKK1 transfection induces a similar effect, thus rescuing the EGFR signalling pathway (figure 5a). This result suggests that NPM1 targets the MAPK pathway downstream of EGFR but upstream of MEK1/2 in order to regulate ERK1/2. Moreover, cotransfection of the caMEKK1 construct with a phEGF-luciferase reporter plasmid also rescues the EGF promoter activity (figure 5b) in LNCaP cells knocked-down for NPM1. These results demonstrate that 1- it is unlikely that NPM1 directly transactivates the EGF promoter in prostate cancer cells. 2- NPM1 may rather stimulate the MAPK signalling pathway to enhance the transcription of the EGF gene. 3- Phosphorylation data on AKT and ERK1/2 strongly suggest that NPM1 acts at the level of MEKK1 or Ras/Raf in this pathway.

## Discussion

NPM1 plays an essential role in cell growth and proliferation. Amongst others, it favours cell cycle progression, ribosome biogenesis and centrosome duplication [5]–[7]. Accordingly, a correlation between increased NPM1 expression levels and tumour progression has been established in a wide set of solid tumours of diverse histological origins such as in gastric- [8], [9], colon [9], kidney [10] or ovary cancers [11]. Nevertheless several studies revealed that, paradoxically, NPM1 is able to both act as a tumour suppressor and as a proto-oncogene during tumourigenesis [12]. On one hand, NPM1 participates to the maintenance of chromosome stability and regulates ARF activity [13] and, on the other hand, NPM1 promotes the inhibition of several tumour suppressors including P53 or Rb, and the activation of the proto-oncogene c-Myc to enhance its transforming activity [14]. Our previous findings showed that the molecular chaperone NPM1 is over-expressed in prostate carcinoma tissue, compared to control adjacent tissue where it stimulates the androgen-dependent transcription [1]. We now wanted to more specifically investigate whether this deregulation of NPM1 expression may act on prostate tumour cells invasive and migration capacities. In this regard, NPM1 was knocked-down in the LNCaP prostate cancer cell lines whose tumour characteristics such as migration, proliferation and invasion capacities are well established. We show that reducing NPM1 expression in LNCaP cells alters the clonogenic and proliferation capacities of these prostate cancer cells as well as their ability to migrate and to invade matrix-containing supports. Accordingly, such cells, when grafted in nude mice, lead to the development of a lower number of also smaller tumours. Conversely, accumulation of NPM1 in LNCaP cells stably expressing an inducible transgene encoding NPM1, increases both clonogenic and proliferation capacities of these cells (figure S2). These results demonstrate that the expression level of NPM1 acts as a controller of the proliferative and of the migration capacities of prostate cancer cells. In accordance with these findings, a recent study by Liu et al. [15] shows that the elevated expression of NPM1 is positively correlated with the formation of metastasis and with poor survival of patients with colon cancer. It is furthermore associated with enhanced migration and invasion properties of colon cancer cells. NPM1 overexpression seems thus to potentiate tumorigenic characteristics of different cancer cell types, and may be an important actor in the evolution of tumours aggressiveness.

NPM1 was described to potentiate the action of the androgen receptor (AR) in prostate cancer cells [1] following a direct interaction with this transcription factor on chromatin. AR, and more widely the androgens signalling, is one of the main pathways that support prostatic tumours growth and metastatic dissemination. Nevertheless, NPM1 inhibition induces similar phenotypes in LNCaP cells that express AR and in PC3 cells that do not express this receptor. It is so unlikely that NPM1 controls prostate cell proliferation and migration via such a mechanism (figure S3).

Besides strongly stimulated androgen signalling, overexpression of the EGF growth factor is also observed in prostate tumours [16]. Further evidences point out that EGF signalling is important for prostate cancer cell proliferation: (i) EGFR is over-expressed in prostate cancer cells [18], [19] (ii) overexpression of EGFR stimulates cell growth *in vitro* and *in vivo*, [20], (iii) in the TRAMP (TRansgenic Adenocarcinoma of the Mouse Prostate) mouse model, the activation of the MAPK pathway involving the ERK1/2 transducers downstream of the EGF/EGFR complex promotes prostate epithelial cell proliferation and tumour progression [21], (iv) ERK1/2 promotes the degradation of extracellular matrix proteins thus favouring tumour invasion [22]. So, it is suggested that these events lead to the establishment of an EGF-dependent autocrine loop which could favour a switch towards a tumour cell-autonomous mechanism and here allow growth factors independent cell growth and proliferation [17]. Such self-regulation loop is characteristic of advanced localized and of therapy resistant prostate cancers. Our results show that LNCaP cells knocked-down for NPM1 accumulate less EGF encoding mRNA than control LNCaP cells. Furthermore, low levels of NPM1 in the cells are associated with a decrease in the EGF promoter activity thus suggesting that NPM1 acts as a positive regulator of EGF gene expression. Although NPM1 is not a transcription factor, this chaperone plays important functions in transcription processes as an actor of chromatin remodelling. In this sense, NPM1 was described as a partner of the acetyltransferase protein p300 or GNC5 in the transcription complex that drives the transcription of the TNFα gene [23]. So, one hypothesis would be that NPM1 can participate to a remodelling complex that enhances transcription at the promoter of the EGF gene.

The increased activity of the EGF/EGFR complex that is associated with prostate tumour progression leads to the activation of different downstream signalling pathways including MEKK/ERK and PI3K/AKT (Phosphoinositide 3-OH Kinase). In NPM1 knocked-down LNCaP cells, we show a reduced activation of the EGF receptor. These findings may well reflect a reduced availability of EGF in these cells, especially as exogenous intake of EGF partially rescues EGFR activation as attested by its phosphorylation status. However, in the same context, activation of ERK1/2 as well as the proliferation and migration capacities of cells are not restored. Moreover, EGF similarly promotes AKT phosphorylation in LNCaP cells independently of their NPM1 levels. This strongly suggests that NPM1 is not necessary for the activation of the PI3K/AKT pathway downstream of the EGFR, but on the contrary may be specifically required for the activation of the MAPK pathway within these cells. Indeed, the transfection of a constitutively active form of MEKK1 in shNPM1 LNCaP cells rescues the phosphorylation status of ERK1/2 and above all, EGF promoter activity. Thus, our data suggest that the major impact of NPM1 seems to occur through the activation of the MAPK pathway, downstream of EGFR, but upstream of MEK1.

Ras proteins are very important in the controlling of the MAPK pathway and are activated following EGF stimulation of EGFR. The spatial organization of these proteins into nanoclusters close to the inner leaflet of the membrane is essential for such activation. Through its ability to shuttle between the nucleus and the cytoplasm, NPM1 exerts some extranuclear functions. Thereby, NPM1 is able to bind and to stabilize K-Ras in an active form, and may thus activate MAPK signalling as described in prostate cancer [24]–[26]. This effect is enhanced after EGF binding on its receptor. We hypothesize that the NPM1 dependent positive regulation of cancer cell migration and invasion secondary to ERK1/2 activation may result from recruitment of K-Ras to the membrane by NPM1.

To conclude, our data demonstrate that NPM1 is involved in the control of prostate cancer cell proliferation and invasion capacities both *in vitro* and *in vivo*. We also show that NPM1 acts as a regulator of the MAPK/ERK pathway and the EGF gene expression, which would explain the change of prostate tumour cell behaviour when NPM1 expression is altered. *A contrario*, when prostate tumour cells display an increased NPM1 expression, one might then expect that it strongly potentiates tumour growth and aggressiveness.

## Supporting Information

Figure S1 **NPM1 knockdown does not induce LNCaP cells apoptosis.** Total proteins from shScr and shNPM1 LNCaP cells cultured in RPMI 1640 10% FBS were analysed by western blotting for PARP (Poly ADP Ribosyl Polymerase) cleavage using a specific anti-PARP antibody (Clone C2-10, 4338-MC-50, Trevigen). As a positive control, shScr LNCaP cells were treated for 24 h with 50 µM cisplatin.
(TIF)

Figure S2 **NPM1 over-expression impacts LNCaP cells three-dimensional growth.** The inducible Flag-NPM1 expressing LNCaP cells were seeded at low confluency on agarose/RPMI 1640 10% FBS for 2 weeks and treated with Doxycycline (1 µg/ml) or vehicle. Number and size of the emerging clones were observed under inverted microscope and photographed. The graph represents the number of cell clones (>50 cells) in the NPM1 overexpression condition, calculated as the mean ± SD of the number of clones counted per field, on 5 random fields, using the ImageJ free software and expressed relatively to the number of clones counted in the control condition, *i.e.* untreated cells. The western blot is representative of three independent experiments and shows the relative accumulation level of the Flag-NPM1 protein using an anti-Flag antibody (F7425, Sigma).
(TIF)

Figure S3 **NPM1 knockdown alters migration and invasion capacities of the PC3 prostate cancer cells.** (**a**) PC-3 cells were transiently transfected using control siRNA (siGFP) or specific NPM1 siRNA (siNPM1). mRNA and protein levels of NPM1 were analysed respectively by RT-qPCR and Western Blotting. (**b**) NPM1 controls migration capacities of PC-3 cells. PC-3 siGFP and siNPM1 cells were plated at confluence in order to create a wound 24 hrs following seeding. Cells were photographed 72 hrs later by inverted microscopy (100× magnification). Histograms show wound areas following quantification with Image J software. (**c**) NPM1 downregulation has an impact on the invasive potential of PC-3 cells. siGFP and siNPM1 transfected PC-3 cells were seeded at confluence in RPMI 1640 with 10%FBS on matrigel in inserts. 48 hours later, cells that invaded the lower of the membrane were fixed and stained with 5% Giemsa and observed at microscope (200× magnification). The data shown are representative of at least three independent triplicates.
(TIF)

Methods S1 **Materials and Methods. Cell culture and transient transfection.**
(DOCX)
