# Supplementary figures and images for: NPM1 Silencing Reduces Tumour Growth and MAPK Signalling in Prostate Cancer Cells
Source: PLoS One. 2014 May 5;9(5):e96293. doi: 10.1371/journal.pone.0096293 (PMC4010470; doi:10.1371/journal.pone.0096293)

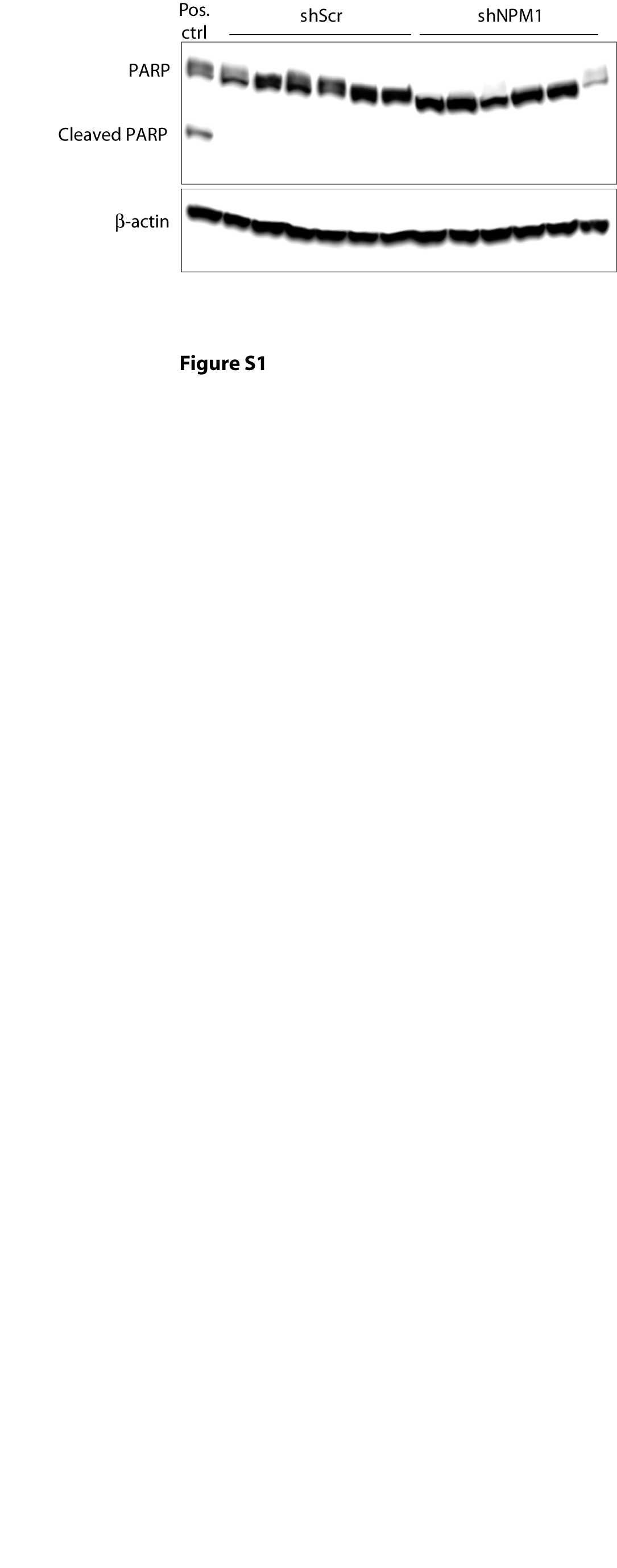

Supplement: Figure S1 — NPM1 knockdown does not induce LNCaP cells apoptosis. Total proteins from shScr and shNPM1 LNCaP cells cultured in RPMI 1640 10% FBS were analysed by western blotting for PARP (Poly ADP Ribosyl Polymerase) cleavage using a specific anti-PARP antibody (Clone C2-10, 4338-MC-50, Trevigen). As a positive control, shScr LNCaP cells were treated for 24 h with 50 µM cisplatin. (TIF) [file pone.0096293.s001.tif]

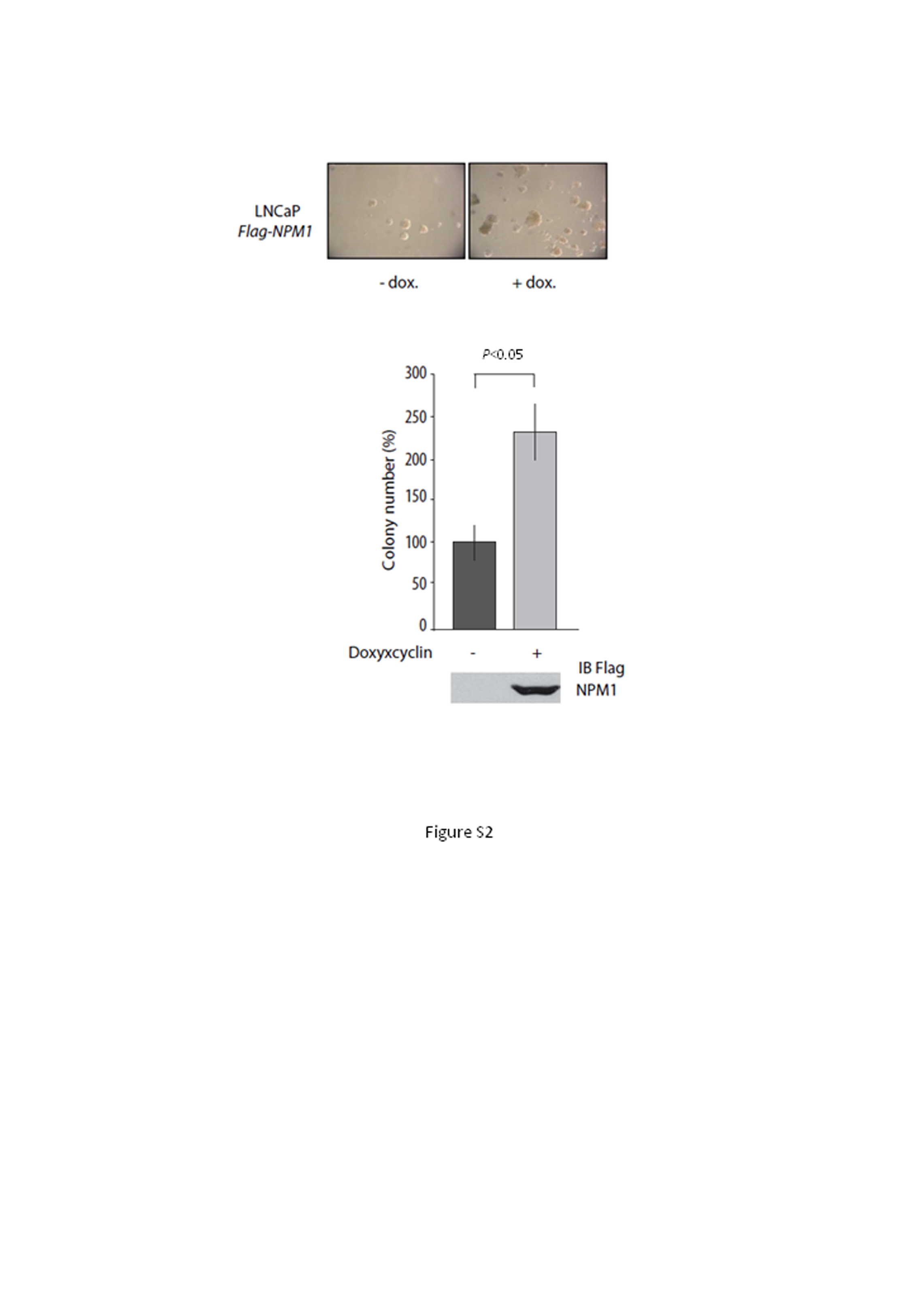

Supplement: Figure S2 — NPM1 over-expression impacts LNCaP cells three-dimensional growth. The inducible Flag-NPM1 expressing LNCaP cells were seeded at low confluency on agarose/RPMI 1640 10% FBS for 2 weeks and treated with Doxycycline (1 µg/ml) or vehicle. Number and size of the emerging clones were observed under inverted microscope and photographed. The graph represents the number of cell clones (>50 cells) in the NPM1 overexpression condition, calculated as the mean ± SD of the number of clones counted per field, on 5 random fields, using the ImageJ free software and expressed relatively to the number of clones counted in the control condition, i.e. untreated cells. The western blot is representative of three independent experiments and shows the relative accumulation level of the Flag-NPM1 protein using an anti-Flag antibody (F7425, Sigma). (TIF) [file pone.0096293.s002.tif]

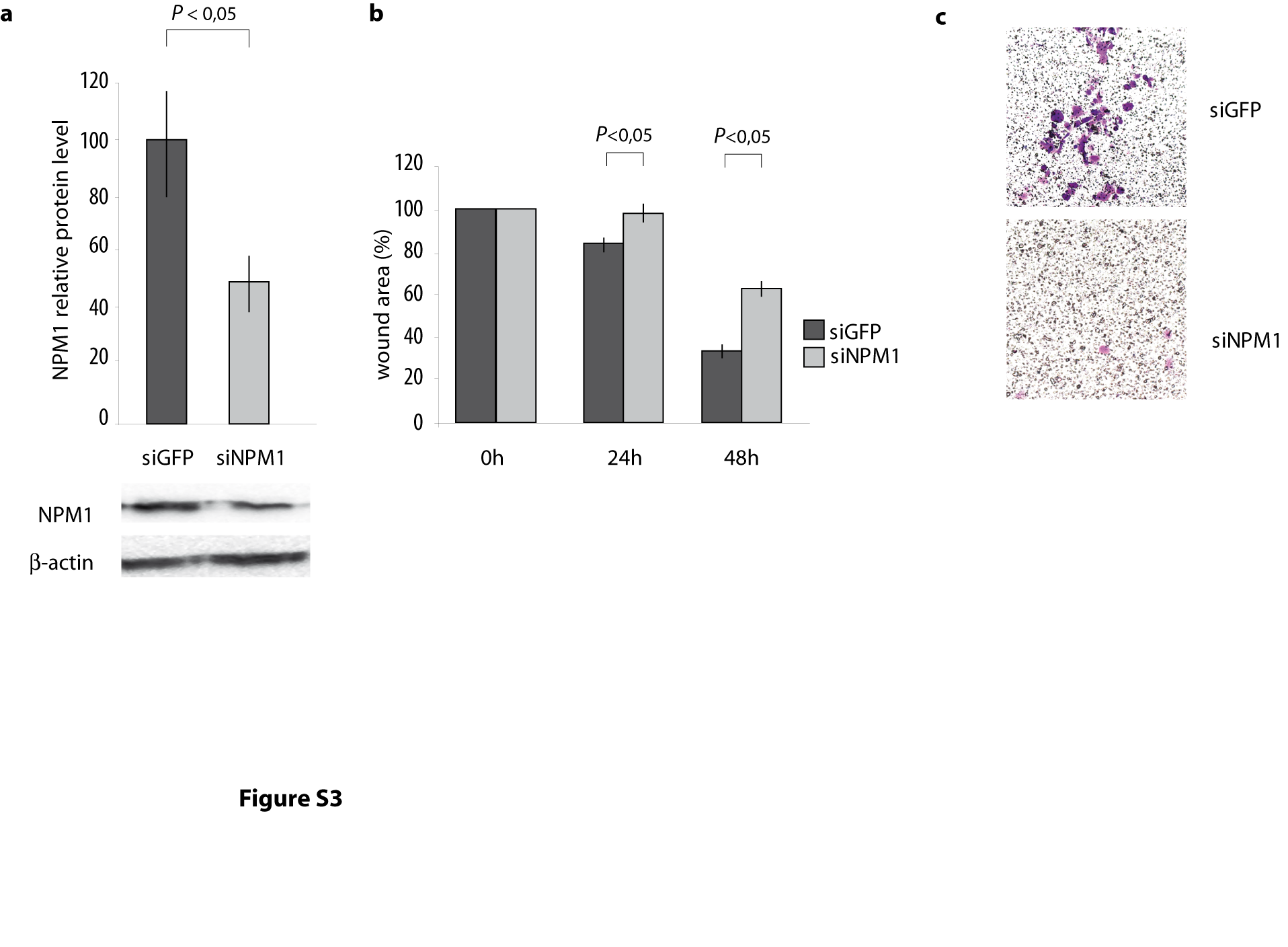

Supplement: Figure S3 — NPM1 knockdown alters migration and invasion capacities of the PC3 prostate cancer cells. (a) PC-3 cells were transiently transfected using control siRNA (siGFP) or specific NPM1 siRNA (siNPM1). mRNA and protein levels of NPM1 were analysed respectively by RT-qPCR and Western Blotting. (b) NPM1 controls migration capacities of PC-3 cells. PC-3 siGFP and siNPM1 cells were plated at confluence in order to create a wound 24 hrs following seeding. Cells were photographed 72 hrs later by inverted microscopy (100× magnification). Histograms show wound areas following quantification with Image J software. (c) NPM1 downregulation has an impact on the invasive potential of PC-3 cells. siGFP and siNPM1 transfected PC-3 cells were seeded at confluence in RPMI 1640 with 10%FBS on matrigel in inserts. 48 hours later, cells that invaded the lower of the membrane were fixed and stained with 5% Giemsa and observed at microscope (200× magnification). The data shown are representative of at least three independent triplicates. (TIF) [file pone.0096293.s003.tif]
