# Supplementary material for: NPM1 Silencing Reduces Tumour Growth and MAPK Signalling in Prostate Cancer Cells
Source: PLoS One. 2014 May 5;9(5):e96293. doi: 10.1371/journal.pone.0096293 (PMC4010470; doi:10.1371/journal.pone.0096293)
Supplement: Methods S1 — Materials and Methods. Cell culture and transient transfection. (DOCX) [file pone.0096293.s004.docx]

**Supporting information**

**Supplementary Materials and Methods**

*Cell culture and transient transfection:*

PC-3 cells were cultured in phenol red Roswell Park Memorial Institute (RPMI) 1640 medium (Gibco, Saint-Aubin, France) supplemented with 10% heat-inactivated fetal bovine serum (FBS) and gentamycin (50µg ml^-1^). For depletion of NPM1, transient transfections were performed using 2 siRNA duplexes: 5’-CCACAGAAAAAAGUAAAACTT-3’ and 5’-UGAUGAAAAUGAGCACCAGTT-3’. GFP siRNA duplex (5’-ACTACCAGCAGAACACCCCTT-3’) was used as a control (MWG, operon). 3.5x10^5^ cells per well were plated in 6-well plates and transfected at the same time by electroporation (Nucleofector II, Lonza) and maintained for 48 to 72 hours in complete medium.

*Wound healing migration assay:*

PC-3 cells were seeded in a 24-well plate and grew to confluence for 24 hours. The monolayer culture was then scrape-wounded with a sterile micropipette tip in order to create a gap of constant width. After removing the cellular debris with Phosphate Buffered Saline 1X (PBS) (Gibco, Saint-Aubin, France), LNCaP cells were exposed continually to various concentrates of NSC348884, an inhibitor of NPM1 (Sigma, Saint-Quentin Fallavier, France). Cells were then grown in RPMI 1640 10% FBS and medium was replaced 12hrs after wound and then every 24hrs. Cells migrated to the wounded region were observed by inverted microscope and photographed at 24hrs, 48hrs and 72hrs (100x magnification). The wound area was measured using the ImageJ free software.

*Boyden Chamber invasion assay:*

The polycarbonate filter (8µm pores) of insert was pre-coated with matrigel as the bottom of the 12-well plate. 3x10^5^ PC-3 cells siGFP or siNPM1 were seeded in FBS free RPMI 1640 in insert in order to be at confluence. RPMI 1640 medium containing 10% FBS was applied to the lower chamber as chemoattractant. Cells were incubated for 24 to 48hrs at 37°C. At the end of the incubation, the cells in the upper surface of the membrane were carefully removed with a cotton swab and the cells that invaded the lower of the membrane were fixed with methanol and stained with 5% Giemsa solution. The invaded cells were observed under microscope (100 or 200x magnification).

*3D growth assays in soft agar:*

FlagNPM1-LNCaP stable clones were obtained following transfection of LNCaP/TR with a pcDNA4T0 plasmid encoding a Flag-NPM1 transgene under the control of a doxycycline inducible promoter, the expression of which is dependent on the presence of doxycycline (1µg/ml) in the culture medium. A 2ml layer of 0.6% low melting agarose (Agarose Sea plaque FMS product low melting, 50101, LONZA, Ozyme, Montigny-le-bretonneux, France) in RPMI 1640, 10%FBS was placed into each well of 6-wells plates. After agar has solidified, 500µl of 0.3% agarose in RPMI 1640, 10%FBS containing 5x10^3^ Flag-NPM1 LNCaP cells were added to each well. The cells were fed in every 3-4 days with RPMI 1640, 10%FBS and treated with 1µg/ml doxycycline or vehicle. The plates were incubated at 37°C, in 5% CO2 for 2 weeks. Colonies were visualized under inverted microscope and photographed (100x magnification). The relative colony number, as reported on the graph, was calculated as the mean ± SD of colonies counted per field, on 5 random fields, using the ImageJ free software relatively to the control condition.
